# (*R*)-2-[(Dimethyl­amino)­meth­yl]-1,1′-bis­(diphenyl­phosphinothio­yl)ferrocene dichloromethane monsolvate

**DOI:** 10.1107/S1600536812022301

**Published:** 2012-05-26

**Authors:** Elisabeth Philippe, Eric Manoury, Jean-Claude Daran

**Affiliations:** a Laboratoire de Chimie de Coordination, CNRS UPR8241, 205 route de Narbonne, Toulouse 31077, France

## Abstract

In the title compound, [Fe(C_20_H_21_NPS)(C_17_H_14_PS)]·CH_2_Cl_2_, both cyclo­penta­dienyl (Cp) rings constituting the ferrocene unit are substituted by a sulfur-protected diphenyl­phosphine. One of the Cp ligands is additionally substituted by a dimethyl­amino­methyl group causing the chirality of the mol­ecule. Surprisingly, although the synthetic procedure yielded the title compound as a racemic mixture, the reported crystal is enanti­omerically pure with the *R* absolute configuration. The dimethyl­amino group is *exo* with respect to the Cp ring. Both diphenyl­thio­phosphine groups are *trans* with respect to the centroid–Fe–centroid direction. Weak intra­molecular C—H⋯S and C—H⋯π inter­actions between symmetry-related mol­ecules are observed. The contribution of the disordered solvent was removed from the refinement using SQUEEZE in *PLATON* [Spek (2009 ▶). *Acta Cryst.* D**65**, 148–155].

## Related literature

For related 1,1′-bis­(diphenyl­thio­phosphino)ferrocene structures, see: Fang *et al.* (1995 ▶); Pilloni *et al.* (1997 ▶) and for a related dimethyl­ethyl­amino­ferrocene structure, see: Mateus *et al.* (2006 ▶). For the chemistry of related ferrocenyl compounds, see: Audin *et al.* (2010 ▶); Debono *et al.* (2010 ▶); Diab *et al.* (2008 ▶); Le Roux *et al.* (2007 ▶); Malacea *et al.* (2006*a* ▶,*b* ▶); Routaboul *et al.* (2005 ▶, 2007 ▶).
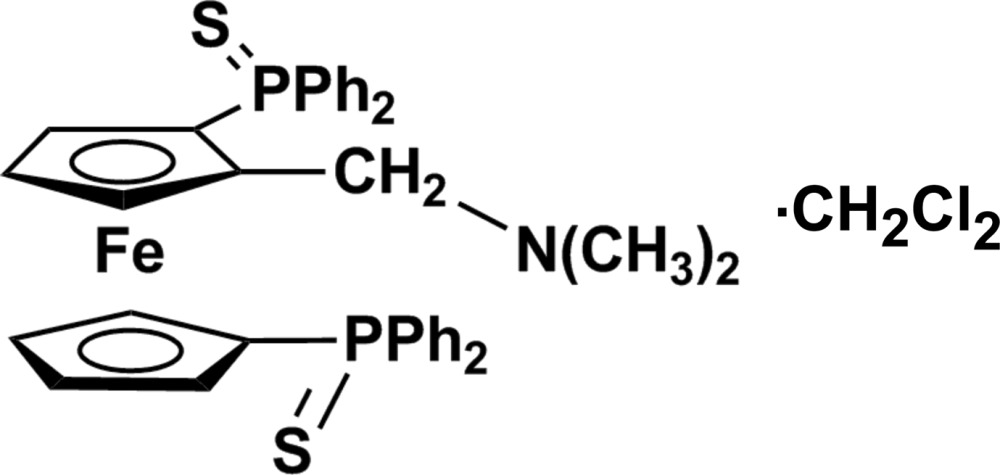


## Experimental

### 

#### Crystal data

[Fe(C_20_H_21_NPS)(C_17_H_14_PS)]·CH_2_Cl_2_
*M* *_r_* = 760.50
Orthorhombic, 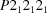

*a* = 8.9493 (3) Å
*b* = 16.8206 (7) Å
*c* = 23.7697 (9) Å
*V* = 3578.1 (2) Å^3^
*Z* = 4
Mo *K*α radiation
μ = 0.81 mm^−1^
*T* = 180 K
0.48 × 0.11 × 0.08 mm

#### Data collection

Bruker APEXII diffractometer
Absorption correction: multi-scan (*SADABS*; Bruker, 2007 ▶) *T*_min_ = 0.841, *T*_max_ = 1.0
61147 measured reflections
7854 independent reflections
7097 reflections with *I* > 2σ(*I*)
*R*_int_ = 0.040

#### Refinement

*R*[*F* ^2^ > 2σ(*F* ^2^)] = 0.042
*wR*(*F* ^2^) = 0.102
*S* = 1.09
7854 reflections
393 parameters
H-atom parameters constrained
Δρ_max_ = 0.63 e Å^−3^
Δρ_min_ = −0.36 e Å^−3^
Absolute structure: Flack (1983 ▶), 3441 Friedel pairs
Flack parameter: 0.043 (16)

### 

Data collection: *APEX2* (Bruker, 2007 ▶); cell refinement: *SAINT* (Bruker, 2007 ▶); data reduction: *SAINT*; program(s) used to solve structure: *SIR97* (Altomare *et al.*, 1999 ▶); program(s) used to refine structure: *SHELXL97* (Sheldrick, 2008 ▶); molecular graphics: *ORTEPIII* (Burnett & Johnson, 1996 ▶) and *ORTEP-3 for Windows* (Farrugia, 1997 ▶); software used to prepare material for publication: *WinGX* (Farrugia, 1999 ▶).

## Supplementary Material

Crystal structure: contains datablock(s) I, global. DOI: 10.1107/S1600536812022301/im2377sup1.cif

Structure factors: contains datablock(s) I. DOI: 10.1107/S1600536812022301/im2377Isup2.hkl

Additional supplementary materials:  crystallographic information; 3D view; checkCIF report

## Figures and Tables

**Table 1:** Hydrogen-bond geometry (Å, °)

*D*—H⋯*A*	*D*—H	H⋯*A*	*D*⋯*A*	*D*—H⋯*A*
C612—H612⋯S6	0.95	2.87	3.367 (3)	114
C113—H113⋯C*T*3^i^	0.95	2.84	3.678 (4)	148

Symmetry code: (i) 
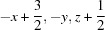
.

**Fig. 1.**
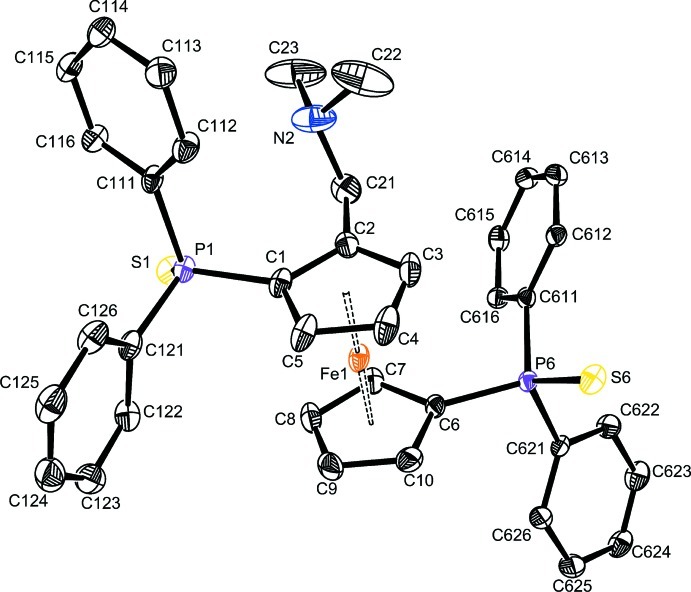
; Molecular view of the title compound with the atom labeling scheme. Displacement ellipsoids are drawn at the 30% probability level. H atoms have been omitted for clarity.

## supplementary crystallographic information

### Comment

Our group has extensively studied the coordination chemistry (Malacea *et al.*, 2006*a*, 2006*b*) and the catalytic properties (Le Roux *et al.*, 2007; Diab *et al.*, 2008; Debono *et al.*, 2010) of various ferrocenyl ligands which can be efficiently synthesized from racemic or enantiomerically pure 2-(diphenylthiophosphino)(hydroxymethyl)ferrocene (Routaboul *et al.*, 2005; Mateus *et al.*, 2006; Routaboul *et al.*, 2007; Le Roux *et al.*, 2007; Audin *et al.*, 2010). The latter may be obtained from 2-(diphenylthiophosphino)(dimethylaminomethyl)ferrocene. This last compound can be efficiently obtained by a one-pot procedure from commercially available dimethylaminomethylferrocene (Mateus *et al.*, 2006). During this synthesis, small amounts of 1,1'-bis(diphenylthiophosphino) 2-dimethylaminomethylferrocene can be observed in the crude materials and isolated by flash chromatography on silicagel. Single crystals suitable for X-ray diffraction analysis could be grown from a dichloromethane solution by slow diffusion of hexane.

In the title compound, both Cp rings constituting the ferrocene unit are substituted by a sulfur protected diphenylphosphine. One of the Cp ligands is additionally substituted by a dimethylaminomethyl group causing the chirality of the molecule. Surprisingly, although the synthetic procedure yielded the title compound as a racemic mixture, the reported crystal is enantiomerically pure with the *R* absolute configuration (Fig. 1). The dimethylamino moiety is *exo* with respect to the Cp ring as already observed for the related 2-(diphenylthiophophino)-dimethylaminomethylferrocene (Mateus *et al.*, 2006). The C2—C21—N2 group is bent with respect to the Cp ring making a dihedral angle of 73.8 (3)° and the two methyl groups have rotated around the C21—N2 bond from the idealized bisecting position to minimize the interactions with the corresponding C111—C116 phenyl ring. The two diphenylthiophosphine moieties are *trans* with respect to the Ct1—Fe—Ct2 centroid direction (Ct1 and Ct2 being the centroids of the C1—C5 and C6—C10 Cp rings, respectively) as it was also observed in the molecular structure of related 1,1-(bisdiphenylthiophosphino)ferrocene (Fang *et al.*,1995; Pilloni *et al.*, 1997). The P1—Ct1—Ct2—P6 torsion angle is 146.75 (2)°.

The two Cp rings are eclipsed with a twist angle of 0.8 (2)°. There is a weak intramolecular C—H···S interaction (Table 1). Weak intramolecular C—H···S and C—H···π interactions between symmetry related molecules are observed (Table 1).

### Experimental

In a Schlenk tube, were dissolved, under argon, 13.5 g (55.6 mmol) of *N*,*N*-dimethylaminomethylferrocene in 80 ml of dry diethylether. The solution was cooled down to -78°C and 42 ml (67.1 mmol) of a 1.65*M n*-BuLi solution in hexane were added dropwise. The solution was then stirred 3 h at RT. After cooling back to -78°C again, 27 ml (150 mmol) of freshly distilled chlorodiphenylphosphine were added dropwise. After stirring overnight at RT, water was added slowly under argon. The aqueous phase was then extracted by three fractions of dichloromethane under argon. The organic solutions were dried with sodium sulfate. After evaporation of the solvents, the crude material was dissolved, under argon, in 400 ml of dry dichloromethane in a Schlenk tube. 10.2 g of sulfur (318 mmol) was then added and the solution was kept at reflux for 2 h. The crude material was purified by flash chromatography on silica with pentane then ether as eluent to yield two yellow fractions (first fraction: 0.45 g of 1,1'-bis(diphenylthiophosphino) 2-dimethylaminomethylferrocene (1.2%); second fraction: 23.2 g of 2- (diphenylthiophosphino)dimethylaminomethylferrocene (91%)).

### Refinement

All H atoms attached to C atoms were fixed geometrically and treated as riding with C—H = 0.95 Å (aromatic), 0.98 Å (methyl), 0.99 Å (methylene) with *U*_iso_(H) = 1.2*U*_eq_(aromatic,methylene) and *U*_iso_(H) = 1.5*U*_eq_(methyl).

Some residual electron densities were difficult to modelize and therefore the SQUEEZE function of *PLATON* (Spek, 2009) was used to eliminate the contribution of the electron density in the solvent region from the intensity data, and the solvent-free model was employed for the final refinement. There are two cavities of 246 Å^3^ per unit cell. *PLATON* estimated that each cavity contains 82 electrons which may correspond to two solvent molecules of dichloromethane as suggested by chemical analyses.

The dimethylamino moiety displays rather large ellipsoids however attempts to modelize a disordered model failed and thus these large ellipsoids reflect rather thermal motion than disorder.

### Crystal data

**Table:**

[Fe(C_20_H_21_NPS)(C_17_H_14_PS)]·CH_2_Cl_2_	*F*(000) = 1576
*M**_r_* = 760.50	*D*_x_ = 1.412 Mg m^−^^3^
Orthorhombic, *P*2_1_2_1_2_1_	Mo *K*α radiation, λ = 0.71073 Å
Hall symbol: P 2ac 2ab	Cell parameters from 9878 reflections
*a* = 8.9493 (3) Å	θ = 2.4–26.6°
*b* = 16.8206 (7) Å	µ = 0.81 mm^−^^1^
*c* = 23.7697 (9) Å	*T* = 180 K
*V* = 3578.1 (2) Å^3^	Needle, yellow
*Z* = 4	0.48 × 0.11 × 0.08 mm

### Data collection

**Table:**

Bruker APEXII diffractometer	7854 independent reflections
Radiation source: fine-focus sealed tube	7097 reflections with *I* > 2σ(*I*)
Graphite monochromator	*R*_int_ = 0.040
ω and φ scans	θ_max_ = 27.1°, θ_min_ = 2.1°
Absorption correction: multi-scan (*SADABS*; Bruker, 2007)	*h* = −11→11
*T*_min_ = 0.841, *T*_max_ = 1.0	*k* = −21→20
61147 measured reflections	*l* = −29→30

### Refinement

**Table:**

Refinement on *F*^2^	Secondary atom site location: difference Fourier map
Least-squares matrix: full	Hydrogen site location: inferred from neighbouring sites
*R*[*F*^2^ > 2σ(*F*^2^)] = 0.042	H-atom parameters constrained
*wR*(*F*^2^) = 0.102	*w* = 1/[σ^2^(*F*_o_^2^) + (0.0472*P*)^2^ + 2.2997*P*] where *P* = (*F*_o_^2^ + 2*F*_c_^2^)/3
*S* = 1.09	(Δ/σ)_max_ = 0.001
7854 reflections	Δρ_max_ = 0.63 e Å^−^^3^
393 parameters	Δρ_min_ = −0.36 e Å^−^^3^
0 restraints	Absolute structure: Flack (1983), 3441 Friedel pairs
Primary atom site location: structure-invariant direct methods	Flack parameter: 0.043 (16)

### Special details

**Table:**

Experimental. NMR of 1,1'-bis(diphenylthiophosphino) 2-dimethylaminomethylferrocene 1H NMR ((p.p.m.), δ CDCl3): 7.7 (2*H*, m: Ph); 7.72–7.60 (6*H*, m: Ph); 7.55–7.40 (8*H*, m: Ph); 7.40–7.30 (4*H*, m: Ph); 4.97 (1*H*, br s: Cp); 4.89 (1*H*, br s: Cp); 4.78 (1*H*, br s: Cp); 4.54 (1*H*, br s: Cp); 4.48 (1*H*, br s: Cp); 4.25 (1*H*, br s: Cp); 3.97 (1*H*, br d(AB), J=10 Hz: CH2); 3.70 (1*H*, br s: Cp); 2.91 (1*H*, br d(AB), J=10 Hz: CH2); 1.88 (6*H*, s: CH3). 13 C NMR (δ (p.p.m.), CDCl3): 134.26 (d, JP—C=87.0 Hz: quat. Ph); 134.22 (d, JP—C=88.3 Hz: quat. Ph); 134.0 (d, JP—C=87.0 Hz: quat. Ph); 133.3 (d, JP—C=85.9 Hz: quat. Ph); 132.0 (d, JP—C=10.7 Hz: Ph); 131.9 (d, JP—C=10.5 Hz: Ph);131.7 (d, JP—C=10.8 Hz: Ph); 131.41 (s, Ph); 131.37 (d, JP—C=12.5 Hz: Ph); 131.3 (s, Ph); 131.2 (d, JP—C=2.9 Hz: Ph); 131.03 (d, JP—C=2.5 Hz: Ph); 128.4 (d, JP—C=12.5 Hz: Ph); 128.2 (d, JP—C=12.5 Hz: Ph); 128.0 (d, JP—C=12.4 Hz: Ph); 127.9 (d, JP—C=12.7 Hz: Ph); 77.9 (br s: Cp); 76.8 (d, JP—C=10.1 Hz: Cp); 76.0 (d, JP—C=12.4 Hz: Cp); 75.8 (d, JP—C=94 Hz: quat Cp); 75.6 (d, JP—C= 9.9 Hz: Cp); 75.3 (d, JP—C=12.3 Hz: Cp); 73.9 (d, JP—C=12.1 Hz: Cp); 72.5 (br d, JP—C=8 Hz: Cp); 56.0 (CH2); 44.5 (N—CH3). 31P NMR (δ (p.p.m.), CDCl3): 40.95; 40,56.
Geometry. All e.s.d.'s (except the e.s.d. in the dihedral angle between two l.s. planes) are estimated using the full covariance matrix. The cell e.s.d.'s are taken into account individually in the estimation of e.s.d.'s in distances, angles and torsion angles; correlations between e.s.d.'s in cell parameters are only used when they are defined by crystal symmetry. An approximate (isotropic) treatment of cell e.s.d.'s is used for estimating e.s.d.'s involving l.s. planes.
Refinement. Refinement of *F*^2^ against ALL reflections. The weighted *R*-factor *wR* and goodness of fit *S* are based on *F*^2^, conventional *R*-factors *R* are based on *F*, with *F* set to zero for negative *F*^2^. The threshold expression of *F*^2^ > σ(*F*^2^) is used only for calculating *R*-factors(gt) *etc*. and is not relevant to the choice of reflections for refinement. *R*-factors based on *F*^2^ are statistically about twice as large as those based on *F*, and *R*- factors based on ALL data will be even larger.

### Fractional atomic coordinates and isotropic or equivalent isotropic displacement parameters (Å^2^)

**Table:**

	*x*	*y*	*z*	*U*_iso_*/*U*_eq_	Occ. (<1)
Fe1	0.48389 (4)	0.00564 (3)	0.606968 (17)	0.03074 (11)	
P1	0.23725 (8)	0.02734 (5)	0.71861 (3)	0.02852 (17)	
P6	0.73306 (8)	0.07876 (5)	0.50958 (3)	0.02409 (16)	
S1	0.13593 (10)	0.12190 (5)	0.68974 (4)	0.0406 (2)	
S6	0.89329 (9)	0.00330 (5)	0.52544 (3)	0.03778 (19)	
N2	0.5450 (5)	0.1520 (2)	0.75679 (14)	0.0686 (11)	
C1	0.4171 (3)	0.0049 (2)	0.68857 (12)	0.0325 (7)	
C2	0.5410 (3)	0.0577 (2)	0.68079 (13)	0.0362 (8)	
C3	0.6606 (3)	0.0106 (3)	0.66053 (13)	0.0494 (10)	
H3	0.7580	0.0300	0.6525	0.059*	
C4	0.6152 (4)	−0.0689 (2)	0.65397 (15)	0.0462 (9)	
H4	0.6740	−0.1116	0.6400	0.055*	
C5	0.4652 (4)	−0.0730 (2)	0.67230 (14)	0.0417 (8)	
H5	0.4059	−0.1198	0.6736	0.050*	
C6	0.5518 (3)	0.04440 (19)	0.52950 (12)	0.0287 (7)	
C7	0.4279 (3)	0.0909 (2)	0.54986 (13)	0.0335 (7)	
H7	0.4281	0.1465	0.5567	0.040*	
C8	0.3061 (3)	0.0388 (2)	0.55776 (14)	0.0419 (9)	
H8	0.2093	0.0541	0.5702	0.050*	
C9	0.3500 (4)	−0.0390 (2)	0.54446 (15)	0.0464 (9)	
H9	0.2893	−0.0852	0.5468	0.056*	
C10	0.5022 (4)	−0.0362 (2)	0.52682 (13)	0.0400 (7)	
H10	0.5607	−0.0804	0.5153	0.048*	
C21	0.5416 (4)	0.1420 (2)	0.69626 (16)	0.0479 (9)	
H21A	0.6301	0.1681	0.6795	0.057*	
H21B	0.4511	0.1680	0.6809	0.057*	
C22	0.6886 (8)	0.1248 (4)	0.7806 (3)	0.119 (3)	
H22A	0.7709	0.1537	0.7626	0.178*	
H22B	0.7003	0.0677	0.7738	0.178*	
H22C	0.6899	0.1350	0.8212	0.178*	
C23	0.5062 (9)	0.2326 (3)	0.7734 (2)	0.111 (3)	
H23A	0.5025	0.2360	0.8146	0.166*	
H23B	0.4082	0.2465	0.7578	0.166*	
H23C	0.5816	0.2697	0.7591	0.166*	
C111	0.2708 (3)	0.03238 (19)	0.79388 (12)	0.0310 (6)	
C112	0.3919 (3)	−0.0059 (2)	0.81862 (13)	0.0402 (7)	
H112	0.4579	−0.0366	0.7961	0.048*	
C113	0.4166 (4)	0.0005 (3)	0.87580 (14)	0.0468 (8)	
H113	0.5002	−0.0254	0.8923	0.056*	
C114	0.3207 (4)	0.0441 (2)	0.90904 (15)	0.0482 (9)	
H114	0.3378	0.0483	0.9484	0.058*	
C115	0.1988 (4)	0.0818 (2)	0.88471 (14)	0.0446 (8)	
H115	0.1323	0.1119	0.9074	0.053*	
C116	0.1741 (4)	0.0757 (2)	0.82769 (13)	0.0362 (7)	
H116	0.0901	0.1015	0.8114	0.043*	
C121	0.1326 (3)	−0.06366 (19)	0.70677 (13)	0.0327 (7)	
C122	0.0559 (4)	−0.0718 (2)	0.65611 (15)	0.0382 (8)	
H122	0.0545	−0.0291	0.6300	0.046*	
C123	−0.0181 (4)	−0.1416 (2)	0.64376 (17)	0.0473 (9)	
H123	−0.0661	−0.1477	0.6084	0.057*	
C124	−0.0227 (5)	−0.2024 (2)	0.68224 (19)	0.0554 (10)	
H124	−0.0775	−0.2494	0.6742	0.067*	
C125	0.0515 (5)	−0.1951 (2)	0.73201 (18)	0.0531 (10)	
H125	0.0490	−0.2376	0.7583	0.064*	
C126	0.1312 (4)	−0.1260 (2)	0.74514 (15)	0.0437 (8)	
H126	0.1836	−0.1218	0.7798	0.052*	
C611	0.7596 (3)	0.17580 (17)	0.54128 (11)	0.0254 (6)	
C612	0.8712 (3)	0.18721 (19)	0.58077 (12)	0.0294 (6)	
H612	0.9324	0.1439	0.5920	0.035*	
C613	0.8938 (3)	0.2616 (2)	0.60395 (14)	0.0384 (7)	
H613	0.9703	0.2691	0.6312	0.046*	
C614	0.8055 (4)	0.3256 (2)	0.58777 (13)	0.0386 (8)	
H614	0.8216	0.3767	0.6037	0.046*	
C615	0.6936 (4)	0.3142 (2)	0.54805 (14)	0.0362 (7)	
H615	0.6327	0.3578	0.5370	0.043*	
C616	0.6703 (3)	0.24041 (18)	0.52467 (12)	0.0302 (6)	
H616	0.5940	0.2331	0.4974	0.036*	
C621	0.7209 (3)	0.09785 (17)	0.43468 (11)	0.0251 (6)	
C622	0.8041 (4)	0.1585 (2)	0.41042 (13)	0.0357 (7)	
H622	0.8578	0.1947	0.4335	0.043*	
C623	0.8085 (4)	0.1661 (2)	0.35233 (14)	0.0438 (9)	
H623	0.8661	0.2075	0.3359	0.053*	
C624	0.7316 (4)	0.1151 (2)	0.31833 (13)	0.0414 (8)	
H624	0.7366	0.1206	0.2786	0.050*	
C625	0.6461 (4)	0.0553 (2)	0.34214 (14)	0.0427 (8)	
H625	0.5918	0.0198	0.3187	0.051*	
C626	0.6398 (3)	0.0471 (2)	0.39988 (13)	0.0359 (7)	
H626	0.5796	0.0065	0.4160	0.043*	
CT1	0.5398	−0.0138	0.6712	0.010*	0.00
CT2	0.4276	0.0198	0.5417	0.010*	0.00
CT3	0.7251	0.1066	0.3763	0.010*	0.00

### Atomic displacement parameters (Å^2^)

**Table:**

	*U*^11^	*U*^22^	*U*^33^	*U*^12^	*U*^13^	*U*^23^
Fe1	0.0273 (2)	0.0402 (3)	0.0247 (2)	0.00255 (18)	0.00526 (16)	0.01199 (19)
P1	0.0269 (3)	0.0326 (4)	0.0260 (4)	0.0068 (3)	0.0028 (3)	0.0074 (3)
P6	0.0225 (3)	0.0311 (4)	0.0186 (3)	0.0000 (3)	0.0010 (3)	0.0037 (3)
S1	0.0450 (5)	0.0375 (5)	0.0393 (5)	0.0162 (4)	−0.0074 (4)	0.0079 (4)
S6	0.0392 (4)	0.0435 (5)	0.0306 (4)	0.0150 (4)	0.0001 (3)	0.0075 (4)
N2	0.111 (3)	0.052 (2)	0.0427 (19)	−0.009 (2)	−0.021 (2)	0.0029 (16)
C1	0.0314 (14)	0.0435 (18)	0.0228 (14)	0.0099 (14)	0.0040 (11)	0.0127 (15)
C2	0.0288 (15)	0.056 (2)	0.0233 (15)	−0.0050 (14)	−0.0038 (12)	0.0097 (14)
C3	0.0232 (14)	0.096 (3)	0.0294 (16)	0.0046 (18)	0.0022 (12)	0.014 (2)
C4	0.0409 (19)	0.055 (2)	0.042 (2)	0.0176 (18)	0.0139 (16)	0.0223 (17)
C5	0.0449 (19)	0.0435 (19)	0.0366 (18)	0.0204 (16)	0.0156 (15)	0.0192 (15)
C6	0.0274 (14)	0.0397 (18)	0.0190 (14)	−0.0062 (12)	0.0039 (11)	0.0055 (13)
C7	0.0269 (14)	0.047 (2)	0.0269 (15)	0.0016 (13)	−0.0022 (12)	0.0104 (14)
C8	0.0242 (14)	0.070 (3)	0.0315 (18)	−0.0044 (15)	−0.0011 (13)	0.0151 (17)
C9	0.0457 (19)	0.058 (2)	0.0356 (19)	−0.0232 (18)	0.0044 (15)	0.0052 (17)
C10	0.0461 (19)	0.0458 (19)	0.0281 (16)	−0.0050 (16)	0.0030 (14)	−0.0014 (14)
C21	0.053 (2)	0.049 (2)	0.042 (2)	−0.0101 (17)	−0.0031 (17)	0.0139 (17)
C22	0.142 (6)	0.097 (4)	0.117 (5)	−0.001 (4)	−0.085 (5)	0.003 (4)
C23	0.211 (8)	0.043 (3)	0.078 (4)	−0.018 (4)	−0.048 (5)	−0.009 (2)
C111	0.0295 (14)	0.0384 (17)	0.0251 (14)	0.0022 (13)	0.0053 (11)	0.0100 (12)
C112	0.0368 (15)	0.056 (2)	0.0275 (15)	0.0090 (17)	0.0019 (12)	0.0102 (16)
C113	0.0436 (17)	0.063 (2)	0.0338 (17)	0.0012 (18)	−0.0049 (14)	0.0122 (18)
C114	0.053 (2)	0.065 (2)	0.0275 (17)	−0.0119 (19)	0.0057 (15)	0.0090 (16)
C115	0.0446 (18)	0.056 (2)	0.0331 (18)	0.0014 (16)	0.0114 (15)	−0.0032 (16)
C116	0.0375 (16)	0.0403 (18)	0.0307 (16)	0.0070 (14)	0.0034 (13)	0.0024 (14)
C121	0.0307 (15)	0.0344 (17)	0.0331 (16)	0.0113 (13)	0.0077 (12)	0.0060 (13)
C122	0.0312 (16)	0.043 (2)	0.0400 (19)	0.0041 (14)	−0.0006 (13)	0.0139 (16)
C123	0.043 (2)	0.046 (2)	0.053 (2)	0.0045 (17)	−0.0012 (17)	−0.0062 (17)
C124	0.056 (2)	0.042 (2)	0.068 (3)	−0.0078 (18)	0.016 (2)	−0.0063 (19)
C125	0.072 (3)	0.032 (2)	0.055 (2)	0.0017 (18)	0.024 (2)	0.0088 (17)
C126	0.058 (2)	0.0350 (18)	0.0384 (19)	0.0098 (17)	0.0115 (16)	0.0066 (15)
C611	0.0224 (13)	0.0321 (15)	0.0216 (13)	−0.0020 (12)	0.0030 (11)	0.0028 (11)
C612	0.0263 (14)	0.0411 (18)	0.0207 (14)	−0.0001 (13)	0.0015 (11)	0.0030 (12)
C613	0.0309 (15)	0.058 (2)	0.0269 (16)	−0.0094 (15)	−0.0011 (13)	−0.0027 (15)
C614	0.0397 (18)	0.045 (2)	0.0312 (17)	−0.0076 (15)	0.0041 (13)	−0.0086 (15)
C615	0.0323 (16)	0.0357 (18)	0.0404 (18)	−0.0003 (13)	0.0070 (13)	0.0062 (15)
C616	0.0286 (14)	0.0381 (17)	0.0239 (14)	0.0016 (13)	−0.0012 (12)	0.0007 (13)
C621	0.0233 (13)	0.0324 (16)	0.0197 (13)	0.0063 (11)	0.0023 (10)	0.0007 (11)
C622	0.0410 (18)	0.0376 (18)	0.0285 (16)	−0.0057 (14)	0.0035 (13)	0.0043 (14)
C623	0.050 (2)	0.050 (2)	0.0310 (17)	0.0018 (17)	0.0107 (15)	0.0144 (16)
C624	0.0448 (18)	0.056 (2)	0.0234 (15)	0.0127 (17)	0.0026 (14)	0.0063 (15)
C625	0.0418 (19)	0.059 (2)	0.0277 (17)	−0.0059 (16)	−0.0037 (14)	−0.0033 (15)
C626	0.0334 (15)	0.0462 (19)	0.0281 (16)	−0.0073 (14)	0.0018 (13)	−0.0036 (14)

### Geometric parameters (Å, º)

**Table:**

Fe1—CT1	1.6401 (4)	C23—H23B	0.9800
Fe1—CT2	1.6488 (4)	C23—H23C	0.9800
Fe1—C2	2.027 (3)	C111—C116	1.388 (4)
Fe1—C1	2.030 (3)	C111—C112	1.391 (4)
Fe1—C3	2.032 (3)	C112—C113	1.381 (4)
Fe1—C7	2.037 (3)	C112—H112	0.9500
Fe1—C10	2.038 (3)	C113—C114	1.378 (5)
Fe1—C6	2.046 (3)	C113—H113	0.9500
Fe1—C5	2.046 (3)	C114—C115	1.388 (5)
Fe1—C4	2.050 (3)	C114—H114	0.9500
Fe1—C9	2.051 (4)	C115—C116	1.377 (5)
Fe1—C8	2.052 (3)	C115—H115	0.9500
P1—C1	1.800 (3)	C116—H116	0.9500
P1—C111	1.816 (3)	C121—C126	1.390 (5)
P1—C121	1.816 (3)	C121—C122	1.393 (5)
P1—S1	1.9551 (11)	C122—C123	1.379 (5)
P6—C6	1.786 (3)	C122—H122	0.9500
P6—C621	1.812 (3)	C123—C124	1.373 (6)
P6—C611	1.813 (3)	C123—H123	0.9500
P6—S6	1.9519 (11)	C124—C125	1.362 (6)
N2—C21	1.449 (5)	C124—H124	0.9500
N2—C23	1.454 (6)	C125—C126	1.399 (6)
N2—C22	1.477 (7)	C125—H125	0.9500
C1—C2	1.432 (4)	C126—H126	0.9500
C1—C5	1.433 (5)	C611—C612	1.384 (4)
C2—C3	1.417 (5)	C611—C616	1.406 (4)
C2—C21	1.464 (5)	C612—C613	1.382 (5)
C3—C4	1.406 (6)	C612—H612	0.9500
C3—H3	0.9500	C613—C614	1.389 (5)
C4—C5	1.413 (5)	C613—H613	0.9500
C4—H4	0.9500	C614—C615	1.389 (5)
C5—H5	0.9500	C614—H614	0.9500
C6—C10	1.428 (5)	C615—C616	1.377 (5)
C6—C7	1.440 (4)	C615—H615	0.9500
C7—C8	1.411 (5)	C616—H616	0.9500
C7—H7	0.9500	C621—C622	1.388 (4)
C8—C9	1.403 (6)	C621—C626	1.393 (4)
C8—H8	0.9500	C622—C623	1.387 (5)
C9—C10	1.426 (5)	C622—H622	0.9500
C9—H9	0.9500	C623—C624	1.364 (5)
C10—H10	0.9500	C623—H623	0.9500
C21—H21A	0.9900	C624—C625	1.385 (5)
C21—H21B	0.9900	C624—H624	0.9500
C22—H22A	0.9800	C625—C626	1.381 (5)
C22—H22B	0.9800	C625—H625	0.9500
C22—H22C	0.9800	C626—H626	0.9500
C23—H23A	0.9800		
			
CT1—Fe1—CT2	176.82 (3)	P6—C6—Fe1	127.72 (16)
CT1—Fe1—C2	37.12 (10)	C8—C7—C6	107.6 (3)
CT2—Fe1—C2	146.06 (10)	C8—C7—Fe1	70.39 (18)
CT1—Fe1—C1	36.75 (8)	C6—C7—Fe1	69.67 (17)
CT2—Fe1—C1	144.12 (8)	C8—C7—H7	126.2
C2—Fe1—C1	41.36 (13)	C6—C7—H7	126.2
CT1—Fe1—C3	35.63 (9)	Fe1—C7—H7	125.3
CT2—Fe1—C3	145.24 (9)	C9—C8—C7	109.4 (3)
C2—Fe1—C3	40.86 (14)	C9—C8—Fe1	70.0 (2)
C1—Fe1—C3	68.34 (12)	C7—C8—Fe1	69.24 (17)
CT1—Fe1—C7	146.69 (10)	C9—C8—H8	125.3
CT2—Fe1—C7	36.50 (10)	C7—C8—H8	125.3
C2—Fe1—C7	109.57 (14)	Fe1—C8—H8	127.1
C1—Fe1—C7	124.64 (13)	C8—C9—C10	107.6 (3)
C3—Fe1—C7	125.46 (16)	C8—C9—Fe1	70.0 (2)
CT1—Fe1—C10	141.04 (11)	C10—C9—Fe1	69.07 (19)
CT2—Fe1—C10	36.33 (10)	C8—C9—H9	126.2
C2—Fe1—C10	159.80 (13)	C10—C9—H9	126.2
C1—Fe1—C10	156.23 (14)	Fe1—C9—H9	126.3
C3—Fe1—C10	122.50 (14)	C9—C10—C6	108.4 (3)
C7—Fe1—C10	68.88 (14)	C9—C10—Fe1	70.1 (2)
CT1—Fe1—C6	144.19 (8)	C6—C10—Fe1	69.84 (19)
CT2—Fe1—C6	36.65 (8)	C9—C10—H10	125.8
C2—Fe1—C6	124.52 (12)	C6—C10—H10	125.8
C1—Fe1—C6	161.71 (14)	Fe1—C10—H10	125.8
C3—Fe1—C6	108.63 (12)	N2—C21—C2	111.3 (3)
C7—Fe1—C6	41.32 (12)	N2—C21—H21A	109.4
C10—Fe1—C6	40.94 (13)	C2—C21—H21A	109.4
CT1—Fe1—C5	35.88 (12)	N2—C21—H21B	109.4
CT2—Fe1—C5	141.48 (12)	C2—C21—H21B	109.4
C2—Fe1—C5	69.07 (15)	H21A—C21—H21B	108.0
C1—Fe1—C5	41.15 (14)	N2—C22—H22A	109.5
C3—Fe1—C5	67.34 (16)	N2—C22—H22B	109.5
C7—Fe1—C5	160.11 (13)	H22A—C22—H22B	109.5
C10—Fe1—C5	119.54 (15)	N2—C22—H22C	109.5
C6—Fe1—C5	155.96 (13)	H22A—C22—H22C	109.5
CT1—Fe1—C4	36.47 (11)	H22B—C22—H22C	109.5
CT2—Fe1—C4	140.93 (11)	N2—C23—H23A	109.5
C2—Fe1—C4	69.38 (15)	N2—C23—H23B	109.5
C1—Fe1—C4	69.18 (13)	H23A—C23—H23B	109.5
C3—Fe1—C4	40.30 (17)	N2—C23—H23C	109.5
C7—Fe1—C4	159.20 (13)	H23A—C23—H23C	109.5
C10—Fe1—C4	104.61 (15)	H23B—C23—H23C	109.5
C6—Fe1—C4	121.01 (13)	C116—C111—C112	118.9 (3)
C5—Fe1—C4	40.36 (13)	C116—C111—P1	119.5 (2)
CT1—Fe1—C9	141.26 (10)	C112—C111—P1	121.6 (2)
CT2—Fe1—C9	36.16 (10)	C113—C112—C111	120.3 (3)
C2—Fe1—C9	158.85 (14)	C113—C112—H112	119.8
C1—Fe1—C9	121.19 (13)	C111—C112—H112	119.8
C3—Fe1—C9	157.44 (17)	C114—C113—C112	120.4 (3)
C7—Fe1—C9	68.37 (15)	C114—C113—H113	119.8
C10—Fe1—C9	40.83 (14)	C112—C113—H113	119.8
C6—Fe1—C9	68.80 (12)	C113—C114—C115	119.6 (3)
C5—Fe1—C9	105.38 (16)	C113—C114—H114	120.2
C4—Fe1—C9	120.38 (17)	C115—C114—H114	120.2
CT1—Fe1—C8	145.23 (9)	C116—C115—C114	120.2 (3)
CT2—Fe1—C8	35.66 (9)	C116—C115—H115	119.9
C2—Fe1—C8	124.85 (15)	C114—C115—H115	119.9
C1—Fe1—C8	108.53 (12)	C115—C116—C111	120.6 (3)
C3—Fe1—C8	161.71 (18)	C115—C116—H116	119.7
C7—Fe1—C8	40.37 (13)	C111—C116—H116	119.7
C10—Fe1—C8	67.88 (15)	C126—C121—C122	119.2 (3)
C6—Fe1—C8	68.33 (12)	C126—C121—P1	122.6 (3)
C5—Fe1—C8	123.01 (14)	C122—C121—P1	118.1 (2)
C4—Fe1—C8	157.15 (16)	C123—C122—C121	120.3 (3)
C9—Fe1—C8	39.98 (15)	C123—C122—H122	119.9
C1—P1—C111	104.65 (13)	C121—C122—H122	119.9
C1—P1—C121	102.87 (15)	C124—C123—C122	120.4 (4)
C111—P1—C121	106.09 (14)	C124—C123—H123	119.8
C1—P1—S1	116.46 (11)	C122—C123—H123	119.8
C111—P1—S1	112.61 (11)	C125—C124—C123	119.8 (4)
C121—P1—S1	113.08 (10)	C125—C124—H124	120.1
C6—P6—C621	105.26 (14)	C123—C124—H124	120.1
C6—P6—C611	107.47 (14)	C124—C125—C126	121.2 (4)
C621—P6—C611	104.87 (13)	C124—C125—H125	119.4
C6—P6—S6	113.92 (11)	C126—C125—H125	119.4
C621—P6—S6	110.44 (9)	C121—C126—C125	119.0 (4)
C611—P6—S6	114.13 (10)	C121—C126—H126	120.5
C21—N2—C23	112.0 (4)	C125—C126—H126	120.5
C21—N2—C22	111.3 (5)	C612—C611—C616	119.6 (3)
C23—N2—C22	113.1 (5)	C612—C611—P6	120.1 (2)
C2—C1—C5	107.4 (3)	C616—C611—P6	120.3 (2)
C2—C1—P1	127.8 (3)	C613—C612—C611	120.1 (3)
C5—C1—P1	124.6 (3)	C613—C612—H612	120.0
C2—C1—Fe1	69.20 (17)	C611—C612—H612	120.0
C5—C1—Fe1	70.05 (17)	C612—C613—C614	120.5 (3)
P1—C1—Fe1	129.88 (15)	C612—C613—H613	119.7
C3—C2—C1	106.4 (3)	C614—C613—H613	119.7
C3—C2—C21	128.6 (3)	C613—C614—C615	119.5 (3)
C1—C2—C21	124.8 (3)	C613—C614—H614	120.3
C3—C2—Fe1	69.78 (19)	C615—C614—H614	120.3
C1—C2—Fe1	69.44 (17)	C616—C615—C614	120.5 (3)
C21—C2—Fe1	129.5 (2)	C616—C615—H615	119.8
C4—C3—C2	110.6 (3)	C614—C615—H615	119.8
C4—C3—Fe1	70.5 (2)	C615—C616—C611	119.9 (3)
C2—C3—Fe1	69.36 (17)	C615—C616—H616	120.1
C4—C3—H3	124.7	C611—C616—H616	120.1
C2—C3—H3	124.7	C622—C621—C626	118.9 (3)
Fe1—C3—H3	127.0	C622—C621—P6	120.4 (2)
C3—C4—C5	106.6 (3)	C626—C621—P6	120.4 (2)
C3—C4—Fe1	69.17 (19)	C623—C622—C621	119.7 (3)
C5—C4—Fe1	69.69 (19)	C623—C622—H622	120.1
C3—C4—H4	126.7	C621—C622—H622	120.1
C5—C4—H4	126.7	C624—C623—C622	121.2 (3)
Fe1—C4—H4	126.0	C624—C623—H623	119.4
C4—C5—C1	108.9 (3)	C622—C623—H623	119.4
C4—C5—Fe1	69.95 (19)	C623—C624—C625	119.6 (3)
C1—C5—Fe1	68.80 (17)	C623—C624—H624	120.2
C4—C5—H5	125.5	C625—C624—H624	120.2
C1—C5—H5	125.5	C626—C625—C624	120.1 (3)
Fe1—C5—H5	127.3	C626—C625—H625	120.0
C10—C6—C7	106.9 (3)	C624—C625—H625	120.0
C10—C6—P6	125.3 (2)	C625—C626—C621	120.5 (3)
C7—C6—P6	127.8 (2)	C625—C626—H626	119.7
C10—C6—Fe1	69.22 (18)	C621—C626—H626	119.7
C7—C6—Fe1	69.01 (17)		
			
C1—CT1—CT2—C8	0.3 (3)	C5—CT1—CT2—C9	1.8 (2)
C2—CT1—CT2—C7	0.1 (2)	C1—C2—C21—N2	71.2 (4)
C3—CT1—CT2—C6	0.6 (3)	C3—C2—C21—N2	−102.8 (4)
C4—CT1—CT2—C10	1.0 (2)		

### Hydrogen-bond geometry (Å, º)

**Table:**

*D*—H···*A*	*D*—H	H···*A*	*D*···*A*	*D*—H···*A*
C612—H612···S6	0.95	2.87	3.367 (3)	114
C113—H113···C*T*3^i^	0.95	2.84	3.678 (4)	148

Symmetry code: (i) −*x*+3/2, −*y*, *z*+1/2.
